# Repeat Surgery in Chronic Aortic Dissection: A New Technique without Touching the Native Aorta

**DOI:** 10.1055/s-0039-3402071

**Published:** 2020-02-19

**Authors:** Gian Luca Martinelli, Attilio Cotroneo, Valerio Tolva, Felice Armienti, Mario Bobbio, Gabriele Musica, Enrico Visetti, Ugo Filippo Tesler

**Affiliations:** 1 Department of CardioVascular, Clinica San Gaudenzio-Gruppo Policlinico di Monza, Novara, Italy; 2 Department of Vascular Surgery, Policlinico di Monza, Monza, Italy; 3 Department of Radiology, Clinica San Gaudenzio-Gruppo Policlinico di Monza, Novara, Italy; 4 Department of Anesthesia, Clinica San Gaudenzio-Gruppo Policlinico di Monza, Novara, Italy

**Keywords:** aortic dissection, repeat surgery, frozen elephant trunk technique

## Abstract

**Background** Repeat surgery of the chronically dissected aorta following repair of a Type-A acute aortic dissection (AAD) still represents a challenge. The proposed surgical options are as follows: (1) staged procedure with elephant trunk (ET) technique, (2) traditional frozen elephant trunk (FET) intervention, and (3) beating heart cerebral vessel debranching followed by thoracic endovascular aortic repair (TEVAR). However, a marked enlargement of the proximal descending thoracic aorta might make it difficult to perform FET/ET intervention. Furthermore, because in conventional surgery for AAD, a prosthetic graft replacement is generally limited to the ascending aorta, and in repeat surgery, this short Dacron graft rarely provides enough room to allow a beating heart cerebral vessel debranching and obtaining a reliable landing zone for the implantation of a firmly anchored stent graft.

**Methods** We retrospectively reviewed all the five consecutive patients treated in our institution, between 2014 and 2017, for chronic aortic dissection after successful surgical treatment of acute Type-A aortic dissection with graft replacement limited to the ascending aorta. The five patients underwent repair utilizing a modified FET technique with total aortic arch and upper descending aorta exclusion without touching the native dissected aorta.

**Results** No early- or midterm mortality was observed. Mean time interval between the initial and the reoperative procedure was 26 months (range, 3–80 months). No patient had a minor/major neurologic event. Mean circulatory arrest time was 16 minutes (range, 11–25 minutes). Mean follow-up time was 22 months (range, 9–42 months).

**Conclusions** We report our initial experience with a modified FET technique realized by anastomosing the stent graft with the previously implanted ascending aortic graft in Hishimaru's zone 0 and by rerouting all cerebral vessels without “touching” the native chronically dissected aorta. A larger number of patients and a longer follow-up will be required to confirm these initial encouraging results.

## Introduction

During conventional surgery for acute Type-A aortic dissection (AAD), a prosthetic replacement limited to the ascending aorta is generally performed, without cerebral vessel debranching and/or arch replacement. 1 2 Aneurysmal dilatation of the residual dissected aorta may occur, particularly if the intimal tear involved the aortic arch at the time of the initial operation. 3 4 5 The need for reoperation is greater in patients with Marfan's syndrome. 6 For this reason, some patients may require surgical repair of the chronically dissected aortic arch and the descending thoracic aorta.

Staged procedures for management of extensive disease of the remaining thoracic aorta, including the use of the elephant trunk (ET) technique, have been successfully used to treat the aforementioned conditions. 7 8 9 10 . Although these are useful techniques, marked aneurysmal dilatation of the proximal descending thoracic aorta (the usual site for anchoring of the aortic graft) is often present in the setting of chronic aortic dissection (ChAD) marking that dilation may preclude the safe anastomosis of a graft to the aorta in this area. Furthermore, the aggregate mortality and morbidity for the staged procedure may be substantial.

A less invasive approach may be represented by a hybrid approach with cerebral vessel rerouting followed by a TEVAR. But the limited length of the previously implanted ascending aortic graft rarely provides enough room allowing a beating-heart cerebral vessel debranching and the creation of a reliable landing zone for the implantation of a firmly anchored thoracic endovascular stent graft. 11 12 13

In the last few years, many publications have advocated the advantage of the FET technique which combines the cut-and-sew technique at the level of the distal aortic arch with an endovascular stent-graft implantation in the descending thoracic aorta. 14 15 16 17 As FET has evolved, many variations of this procedure have been described. All these procedures have in common that the stent graft is delivered into the open aorta under circulatory arrest and it is sutured, at least partially, into the dissected aortic arch. This intervention is a major surgical procedure, requiring prolonged time on cardiopulmonary bypass (CPB), hypothermic circulatory arrest, and selective cerebral perfusion. The prolonged spinal cord ischemia, associated with the exclusion of several intercostal arteries, increases the risk of paraplegia. In the Bologna experience, Di Eusanio et al 18 described an incidence of 7.4% permanent neurologic dysfunction and 9.0% spinal cord injury. Further, the execution of the distal anastomosis in Hishimaru's zone 3 may be impaired by its distal location and by the frequent poor quality of the dissected aortic wall in this area.

We propose a new technique which preserves all the advantages of FET. Anastomosing the stent graft to the previously implanted ascending aortic graft allows complete exclusion of the aortic arch and of the proximal thoracic descending aorta without “touching” the native chronically dissected aorta. Since 2014, we have employed this technique in five patients without observing any early or midterm mortality or any major neurological complications.

## Materials and Methods

We retrospectively analyzed all consecutive patients treated in our institution between 2014 and 2017 for ChAD after the successful surgical treatment of acute Type-A aortic dissection with graft replacement of the ascending aorta.

Patients in whom cerebral vessels rerouting had been performed at the first operation have been excluded from this report. They were treated by traditional thoracic endovascular aneurysm repair (TEVAR) at the repeat operation. Furthermore, when the previous ascending aorta graft implant was longer than 5 cm, cerebral vessel debranching on beating heart was performed to obtain a reliable landing zone for allowing a stable anchored stent graft for TEVAR. Also, this group of patients was excluded from this report.

The institutional review board approved this study. All patients provided written consent.

Five patients underwent surgical correction of the chronic dissection with our modified FET technique with total aortic arch and upper descending aortic exclusion without touching the native aorta. The same surgeon performed all procedures. The indication for surgery was aneurysmal enlargement of a ChAD involving the aortic arch and the descending thoracic aorta. The choice of this technique was related to the presence of a short previous ascending aortic graft (<5 cm). We used a C-Tag stent graft (C-TAG, WLGore Arizona) of 31 of 34 mm diameter and 20-cm length in all patients.

Patient characteristics are described in Table 1 . Mean age was 52 ± 15 years, and 80% were men. There were two Marfan's patients. All patients were in NYHA class 1 to 2. Mean delay time between first operation for acute aortic dissection and the second operation was 26 months (range, 3–80 months).

**Table 1:** Baseline patient characteristics

Characteristics	*n = 5*	%
Age (y)	52 ± 15	–
Male	4	80
Left ventricular ejection fraction >50%	5	100
NYHA class I–II	5	100
Elective surgery	5	100
Previous surgery for AAD	5	100
Interval between operations (mo)	26 (range, 3–80)	–

Abbreviations: AAD, acute aortic dissection; NYHA, New York Heart Association.

### Surgical Technique

Preoperative computed tomography (CT) of the chest, abdomen, and pelvis is performed to assess surgical strategy and to determine the stent-graft distal landing zone. The selection of the stent graft is based on the diameter of the previously inserted graft in the ascending aorta with an oversizing of 2 to 4 mm. As an example, in a 28 of 30-mm graft in the ascending aorta, we generally insert a 34-mm stent-graft. Such selection proves adequate for stenting the descending aortic aorta.

The first step of the procedure consists of performing an 8-mm PTFE carotid-left subclavian artery bypass, through a left supraclavicular incision. Pressures are monitored in both radial arteries and in the left femoral artery. Cerebral oxymetry is assessed by the noninvasive INVOS system (Covidien, Medtronic Inc., Minneapolis, MN). Next, an echo-guided pig-tail catheter is threaded though the left femoral artery in the true lumen of the dissected aorta until its tip reaches the lumen of the previously implanted ascending aortic graft.

**Fig. 1:**
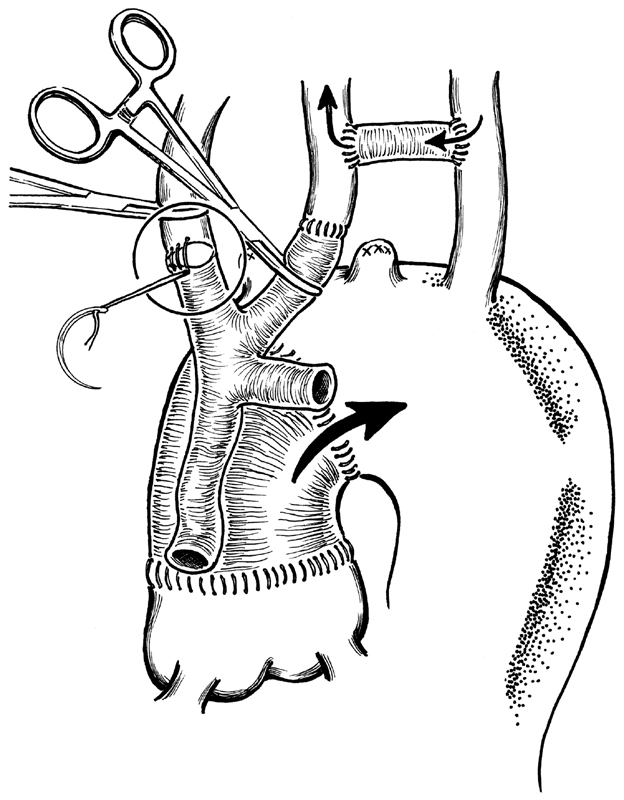
Carotid-left subclavian artery bypass and innominate and left carotid reimplantation on the trifurcated graft. The arrow indicates the direction of blood flow for cerebral vessels perfusion during all the 4 steps of the operation: anterograde flow, on beating heart, guarantee left subclavian artery and left carotid throw the PTFE carotid-left subclavian artery bypass.

Median sternotomy is performed and the incision is extended to the left cervical area for ease of control of the cerebral vessels, particularly of the origin of the left subclavian artery. A trifurcated modified graft with a side-arm perfusion port is selected (Hemashield's 12–8–8 mm) and two of its distal branches are respectively anastomosed to the transected left common carotid and to the innominate artery with the heart beating. 18 During this time, the cerebral vessels are perfused via the native left subclavian artery previously anastomosed to the left carotid artery ( Fig. 1 ). With sequential debranching and reperfusion of the three head vessels, as described by Matalanis et al, 19 complete and rapid cerebral perfusion is ensured.

**Fig. 2:**
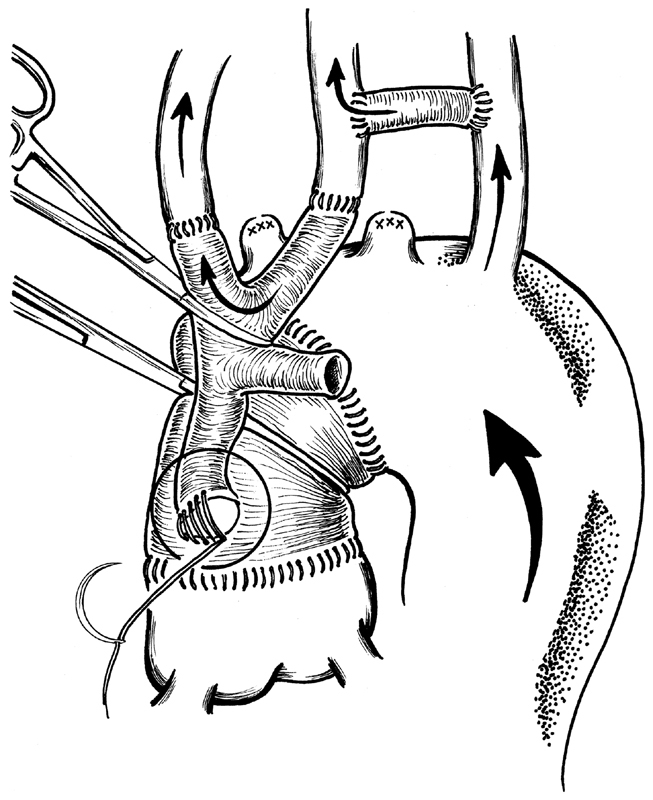
Trifurcated graft anastomosed proximally on the ascending aorta graft on cardiopulmonary bypass with aorta cross clamped to allow an easy proximal reimplantation close to the previous anastomosis. Retrograde flow from cardiopulmonary bypass throw femoral arterial cannulation. Please note, at this time of the operation, left carotid artery and the innominate artery are perfused throw carotid-left subclavian bypass graft.

CPB is then started between the right femoral artery and the right atrium. The ascending aorta is crossclamped and antegrade cardioplegia is infused. At this point, the cerebral vessels are perfused by the CPB in a retrograde fashion through the subclavian artery. The proximal extremity of the trifurcated graft is anastomosed to the graft originally implanted in the ascending aorta ( Fig. 2 ). When this stage is completed, antegrade perfusion of all cerebral vessels is achieved via the cannulated third-arm perfusion port of the trifurcated graft. The native left-subclavian artery is ligated.

An appropriate stent graft is selected and prepared. The patient is cooled to 28°C and CPB is discontinued. Under circulatory arrest, the ascending Dacron graft is opened proximally to the old distal anastomosis. The selected stent graft is prepared. A superstiff wire guide (Lunderquist extra stiff wire guide, Cook Medical LLC, Bloomington, IN) is introduced through the pigtail previously inserted from the left femoral artery and, through this access, the stent graft is deployed in an antegrade fashion under direct vision.

**Fig. 3:**
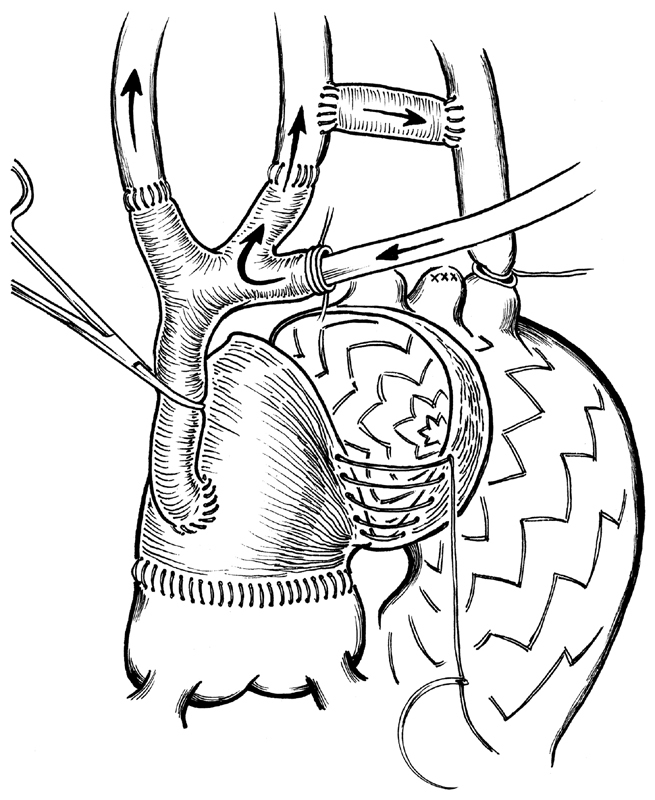
Stent-graft released under circulatory arrest and fixed to the previously implanted ascending aorta graft. During circulatory arrest time, all three cerebral vessels are continuous perfused by a cannula inserted on the trifurcated modified graft.

Once the stent graft is deployed, the stent graft is sutured to the original ascending aorta Dacron graft ( Figs. 3 4 5 ).

**Fig. 4:**
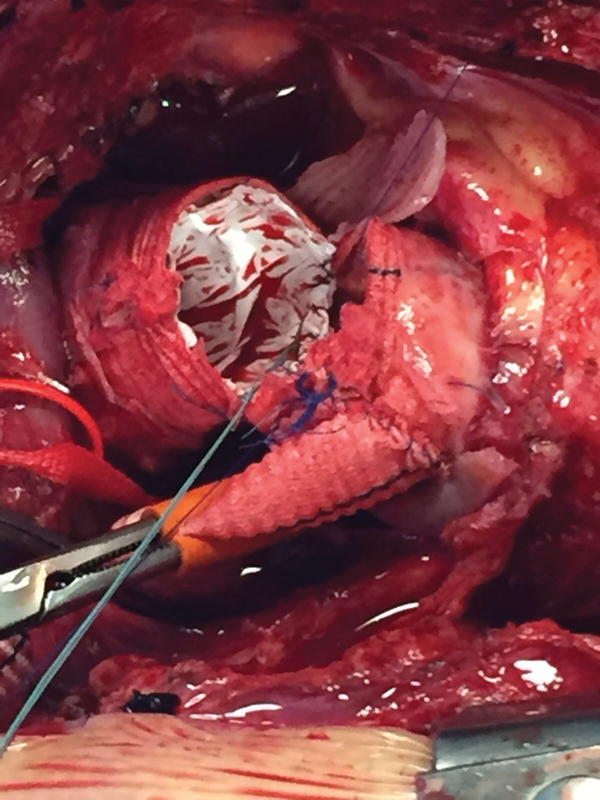
Intraoperative photograph of stent graft sewing over previous implanted ascending aorta Dacron graft. Reproduced with the permission from photographic archives of Clinica San Gaudenzio, Novara.

**Fig. 5:**
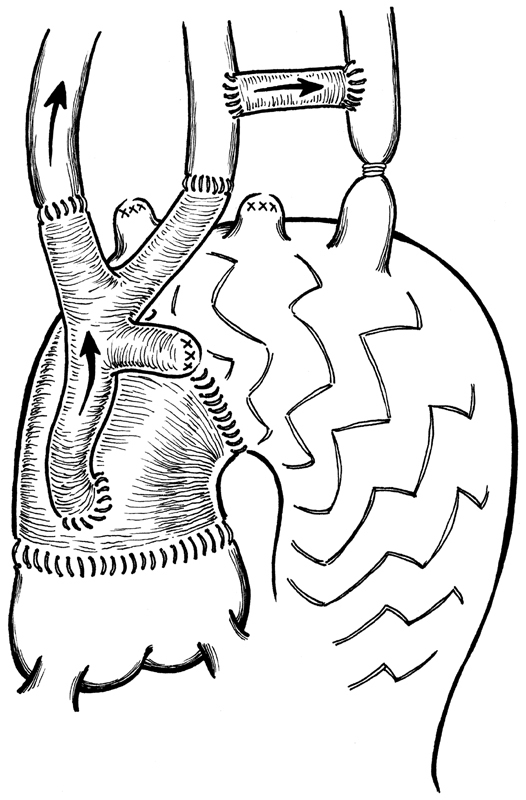
Final result with aortic arch and descending thoracic aorta replacement. At the end of the operation, we remove the clamp on the principal branch of the trifurcated graft and all three cerebral vessels are perfused.

At this point, CPB is restarted. When the rewarming is complete, cardiopulmonary is discontinued.

## Results

There were no intraoperative or in-hospital deaths. Table 2 describes continuous operative data. The postoperative course was uncomplicated for all patients. All patients were extubated on the first postoperative day and discharged within 15 days. No patient had a minor/major neurologic event.

**Table 2:** Continuous operative data

Variables (min)	Mean (range)
Cardiopulmonary bypass	141 (98–171)
Cardiac arrest time	74 (54–93)
Hypothermic circulatory arrest	16 (11–25)
Selective cerebral perfusion	31 (15–80)

Note: selective cerebral perfusion was considered the perfusion of the cerebral vessels by the dedicated trifurcated graft; all three cerebral vessels were perfused.

**Fig. 6:**
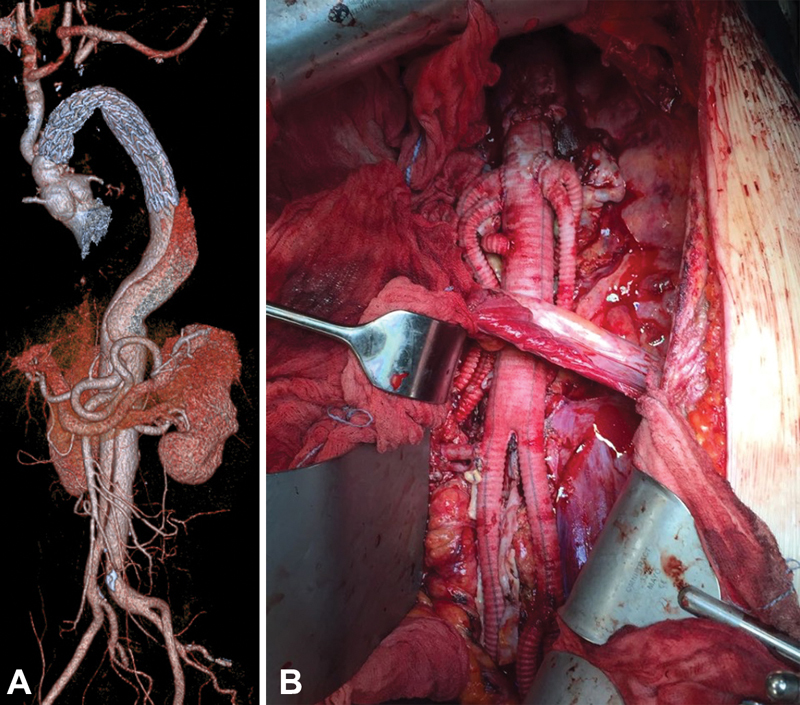
Volume-rendered computed tomography reconstruction in a Marfan patient: **(A)** first step: aortic arch and descending aorta replacement and **(B)** second step: thoracoabdominal aortic replacement.

Follow-up was complete for all patients with a postoperative CT scan ( Fig. 6 ). Mean follow-up time was 22 months (range, 9–42 months). No mortality was observed. Three patients underwent thoracoabdominal surgery within 3 months from the operation. One patient has a Type-II endoleak due to an incomplete ligation of the subclavian artery that was treated successfully by coil embolization.

## Discussion

In patients with chronic dissection, aortic repair is usually performed electively for aneurysmal degeneration of the false lumen. Most of these patients are survivors of De Bakey's Type-1 dissections. After repair of acute Type-A aortic dissection by graft replacement of the ascending aorta, the false lumen distal to the aortic graft remains patent in 70 to 100% of patients. 20 Follow-up has shown that reoperation for aneurysmal dilatation of residual dissected aorta is required in 20 to 30% of patients within the first 5 to 7 years. The need for reoperation is more common in patients with Marfan's syndrome and when the original intimal tear involved the aortic arch at the time of the first operation. 3 4 5 6

For this indication, it is not only important to cover the proximal entry tear; one must also address all-entry tears feeding the aneurysmal segment of the aorta. Most commonly, this aneurysmal degeneration occurs in the distal aortic arch and the proximal descending aorta, making the FET technique particularly well suited for this indication. The rationale for using a stent graft in ChAD is the proximal expansion of the true lumen covering the reentry sites along the proximal descending thoracic aorta, as well as the realization of a satisfactory landing zone, for a possible future distal aortic reoperation.

As FET has evolved, many variations for implantation have been described. 20 21 22 23 Their common feature is that the stent graft is delivered into the open aorta under circulatory arrest and sutured into position. However, this necessitates suturing the stent graft, at least partially, into the dissected aortic arch. Although these are useful techniques, marked aneurysmal dilatation of the proximal descending thoracic aorta, the usual site for attachment of the aortic graft, is often present in the setting of ChAD and may preclude safe anastomosis of a graft to the aorta in this area. Furthermore, all hybrid grafts retain the complexities of surgery and its associated risks, particularly the prolonged ischemic durations of the heart, brain, spinal cord, and viscera. 24 25

We have described a new surgical approach for the treatment of chronic aortic arch and proximal descending aortic dissection following repair of a Type-A acute aortic dissection. The advantage of our technique is that, after releasing the stent graft under circulatory arrest, we secure it to the graft previously implanted in the ascending aorta in without touching the native dissected aorta. The performance of an anastomosis in Hishimaru's zone 0 presents an easy surgical view and an enhanced suture control. Consequently, we have observed fewer hemorrhagic complications with greater hemodynamic stability in the early postoperative period. Other advantages of this technique include the short circulatory arrest time and, thus, a theoretically decreased likelihood of spinal cord damage. In our initial experience, we did not observe any neurologic complications. Another advantage is the avoidance of a potential injury to the recurrent laryngeal nerve.

During conventional surgery for AAD, a short ascending aortic replacement is generally performed, without cerebral vessel debranching and arch replacement. In repeat surgery, this surgical graft rarely provides enough room to perform cerebral vessel debranching on beating heart and to obtain a reliable landing zone for a stable TEVAR. Length of the ascending aorta Dacron graft less than 5 cm was considered a contraindication to performing a total cerebral vessel rerouting on beating heart followed by a TEVAR.

Recently, we have extended this surgical strategy not only for the treatment of ChAD but also for the treatment of arch and proximal descending thoracic aorta aneurysms for one step repair or as a bridge to definitive thoracoabdominal repair.

## Conclusions

This study documents our experience with a small number of patients and a short follow-up period. However, our data suggest that this modified FET technique without “touching” the native dissected aorta constitutes a suitable alternative to a more complex surgery such as the staged ET approach or the classical FET technique with the distal anastomosis in the descending thoracic aorta. Our study may represent a less demanding solution to redo Type-A aortic dissection surgery in which substantial enlargement of the proximal descending thoracic aorta precludes an easy suturing of a prosthetic graft to this segment of the aorta. This new technique is now our preferred method of management. A larger number of patients and longer follow-up will be required to confirm these initial encouraging results.
